# A Neurodynamic Perspective on Musical Enjoyment: The Role of Emotional Granularity

**DOI:** 10.3389/fpsyg.2017.02187

**Published:** 2017-12-13

**Authors:** Nathaniel F. Barrett, Jay Schulkin

**Affiliations:** ^1^Institute for Culture and Society, University of Navarra, Pamplona, Spain; ^2^Department of Neuroscience, Georgetown University, Washington, DC, United States

**Keywords:** music cognition, music and emotion, granularity of emotion, neurodynamical models, musical enjoyment

## Introduction

Musical enjoyment is a nearly universal experience, and yet from a neurocognitive and evolutionary standpoint it presents a conundrum. Why do we respond so powerfully to something apparently without any survival value? A variety of explanations for the evolution of music cognition have been offered (e.g., Wallin et al., 2000; Morley, 2013), nevertheless most current neurocognitive theories of its specifically affective aspects do not posit any specially adapted emotional circuitry (but see Peretz, 2006). Rather, it is assumed that whatever processes are responsible for pleasure and emotion in general—be they subcortical, cortical, or both—are also responsible for the thrills of music. Accordingly, the problem of musical enjoyment is to explain how and why these processes are engaged so effectively by musical stimuli.

While this is a perfectly sensible approach, a major obstacle lies in its path: the paradox of enjoyable sadness in music (Davies, 1994; Levinson, 1997; Garrido and Schubert, 2011; Huron, 2011; Vuoskoski et al., 2011; Kawakami et al., 2013; Taruffi and Koelsch, 2014; Sachs et al., 2015). Music frequently elicits experiences of negative emotions, especially sadness, which we nevertheless find deeply gratifying. For neurocognitive perspectives, the implication of this phenomenon is that normal processes for generating emotional responses are not sufficient to explain musical enjoyment, as something special to music must allow us to enjoy negative emotion. How is musically induced sadness different from “normal” sadness such that the former can be enjoyed while the latter cannot?

To make progress on such questions, in this article we propose a theory of musical enjoyment based on implications of a *neurodynamic* approach to emotion (Pessoa, 2008; Flaig and Large, 2014), which highlights the role of transient patterns of coordinated neural activity spanning multiple regions of the brain. The key advantage of this perspective is its ability to register the possibility that emotional experiences differ not only in kind (happy vs. sad) but also in “granularity,” complexity, or differentiation (Lindquist and Barrett, 2008). Studies indicate that increased emotional granularity functions as a kind of positivity that can meliorate experiences of negative emotions (Smidt and Sudak, 2015). Accordingly, perhaps we can account for enjoyment of negative emotions in music if we can show that these emotions are more finely differentiated than “normal” negative feelings.

Based on this approach, we also propose a general distinction between *pleasure*, defined here as bursts of positively categorized feeling, and *enjoyment*, defined as sustained flows of finely differentiated feeling regardless of emotional categorization (cf. Frederickson, 2002). For this perspective, the categorical meaning of an emotional experience (e.g., happy or sad) is closely related to but separable from its positive or negative affective tone, at least insofar as this tone is influenced by granularity.

## Neurodynamics, emotion, and music

Here “neurodynamic approach” refers to a family of neurocognitive theories that regard transient, large-scale patterns of rhythmically coordinated neural activity as the main vehicles of cognition/emotion (e.g., Freeman, 1997; Bressler and Kelso, 2001, 2016; Varela et al., 2001; Cosmelli et al., 2007; Breakspear and McIntosh, 2011; Sporns, 2011). An especially pertinent feature of this approach is its divergence from traditional notions of functional specialization and localization (Pessoa, 2008, 2014). Broadly speaking, insofar as neurodynamic theories understand cognitive functions as supported by transient, task-specific coalitions rather than stable processing pathways, they tend to affirm the multifunctionality of neural structures at multiple scales (Anderson et al., 2013; see also Hagoort, 2014; Friederici and Singer, 2015). According to this view, which contrasts with the modular approach commonly adopted by computational theories of neural function, the precise functional role of any given neural structure changes according to the context of coordinated neural activity (McIntosh, 2000, 2004; Bressler and McIntosh, 2007). On the other hand, this approach does not herald a return of “equipotentiality,” as it allows for characterizations of the functional “dispositions” of neural structures (Anderson, 2014).

Similarly, with regard to affect and emotion, the neurodynamic perspective can register the importance of distinct structures—e.g., subcortical structures and hedonic “hotspots” (Berridge and Kringelbach, 2015). But it holds that the full range of emotional experience must be understood in terms of continually evolving patterns of globally coordinated neural activity. Thus, while localized structures may have consistent roles in the production of emotional responses, they do not by themselves constitute emotion nor can they be said to govern emotion in any simple way (Flaig and Large, 2014). The point is not just that emotion is the product of the continual interplay of cortical and subcortical dynamics (Panksepp, 2012). Rather the key implication of neurodynamics is that this interplay is constituted by transient patterns of coordinated activity whose dynamic features—especially complexity and continuity—are relevant to the categorization of emotion and affect (cf. Spivey, 2007 on dynamical categorization). Here, we are mainly interested in the possibility that neurodynamic categorizations of emotional content can range in complexity, corresponding to differences of emotional granularity in experience (analogous to the difference between simple and rich color palettes).

It should be noted that this approach encompasses both categorical theories of “basic emotions” (e.g., Panksepp, 1998, 2007) and dimensional theories of “core affect” (Russell, 2003; Barrett et al., 2007). What is essential for present purposes is the way in which the dynamic interplay between subcortical responses and the complex cortical elaboration of emotion (Reybrouck and Eerola, 2017) gives rise to both categorical distinctions (happy vs. sad) *and* differences of granularity (fine vs. coarse).

Neurodynamic approaches are well-established in the field of music cognition (for reviews see Large, 2010; Flaig and Large, 2014). Among the advantages of a neurodynamic approach is its capacity to register the relationship between bodily movement and music (e.g., Large et al., 2015). This relationship has been indicated by numerous studies of sensorimotor involvement in music perception (e.g., Chen et al., 2008) and must be taken into account by any theory of musical experience, as we briefly indicate below.

However, few attempts have been made to understand musical enjoyment from a neurodynamic perspective (see Chapin et al., 2010; Flaig and Large, 2014). A notable exception is William Benzon's groundbreaking treatise (Benzon, 2001), which anticipates the perspective offered here. One reason for this neglect is the challenge of empirical verification: like neurodynamic theories of consciousness (Seth et al., 2006), neurodynamic theories of musical experience are in need of high temporal-resolution data (e.g., from EEG or MEG) that show how relevant characteristics of neural dynamics change during musical experience (see Garrett et al., 2013). For the current proposal, the key challenge is to find and measure just those variations that correspond to differences of emotional granularity or complexity. Until such methods are developed, studies of emotional differentiation in musical experience must turn to the refinement of self-reporting methods (Juslin and Sloboda, 2010).

## The paradox of enjoyable sadness in music

In this section, we consider the challenge posed by deeply gratifying experiences of musically induced sadness (Sachs et al., 2015). Sadness is being used here as representative case, as it seems that other negative emotions—despair, terror, dread—can also be induced and enjoyed through music (Gabrielsson, 2011). Also, we do not mean to claim that music is the only source of enjoyable sadness. Rather, our purpose is to use the case of enjoyable sadness in music to set up a distinction between *pleasure*, defined as bursts of positively categorized feeling, and *enjoyment*, defined as any sustained flow of high-dimensional feeling, regardless of emotional categorization.

First, let us briefly consider other ways of handling the paradox of enjoyable sadness in music. Some have suggested that music can induce both negative and positive affect at once (Larsen and Stastny, 2011). Others have theorized that sadness might be *perceived* in music but is not actually *felt* (Kivy, 1990; Garrido and Schubert, 2011; Kawakami et al., 2013). A third possibility is that sadness is not enjoyed but is compensated for by other positive emotions or by the positive value of the overall experience (Davies, 1994; Huron, 2011). How to decide among these theories?

Part of the problem is that the phenomenological questions raised by enjoyable sadness are subtle and difficult to settle conclusively. Data from self-reporting shows a variety of responses to sad music, including complex emotions that are difficult to put into words (Taruffi and Koelsch, 2014) as well as instances in which sadness is perceived but not felt. Nevertheless, there is ample evidence that music is capable of inducing powerful emotions (Gabrielsson, 2011), including sadness (Vuoskoski and Eerola, 2012), and there is little evidence in support of the idea that musically induced emotions are “less real” than normal emotions (Scherer, 2004). From a physiological standpoint they seem to be identical, evoking the same autonomic responses—chills, elevated heart rate, etc. (Hodges, 2010).

Indeed, musically induced emotions can sometimes feel *more* real insofar as they are more precisely specified than normal emotions. Felix Mendelssohn famously observed that our experience of emotion in music is “too precise for words,” suggesting that music is used not only to induce but also to expand and enrich emotion (Krueger, 2014). In light of this possibility, we believe that the simplest explanation for the popularity of sad music (e.g., Adele's “Someone Like You”) is that people are drawn to the enjoyment of musically enriched negative emotion for its own sake.

While far from exhaustive, we hope this discussion suffices to indicate that the paradox of enjoyable sadness in music is not yet resolved. The emotion of sadness in musical experience can be vividly real in both physiological and subjective senses and yet also thrilling in a way that normal sadness is not. Moreover, the question of enjoyable sadness is only partly explained by musical features that commonly evoke sadness (Guhn et al., 2007) or by evidence that strong experiences of musical emotion are accompanied by the release of dopamine (Salimpoor et al., 2011), as neither explains how an experience can feel sad and enjoyable at once.

## Pleasure and enjoyment

Philosophers and psychologists have long distinguished between sensory pleasures and more fulfilling experiences of enjoyment and happiness (see Berridge and Kringelbach, 2011; Katz, 2016). For example, a distinction between pleasure and enjoyment is widely affirmed in positive psychology (Csikszentmihalyi, 1990; Frederickson, 2002). However, to our knowledge this distinction has not been verified experimentally.

We believe that a neurodynamic approach can help to refine this distinction and to develop testable hypotheses concerning its neural basis. For instance, based on the neurodynamic approach sketched above, it can be theorized that pleasure pertains to positively categorized feelings that are constituted by the momentary, stereotypical effects of subcortical responses on cortical dynamics. Meanwhile enjoyment might be associated with temporally extended patterns of cortical dynamics that are marked by sustained high dimensionality and that can vary independently of subcortical input. This hypothesis is consistent with studies that suggest that rich sensorimotor engagement can give rise to enjoyment without stimulating any of the drives or appetites normally associated with pleasure (Nakamura and Csikszentmihalyi, 2002), but it requires more elaboration in both phenomenological and neurological terms. In short, what is needed is a detailed analysis of interrelated but distinct aspects of positive affect that are frequently lumped together as “positive emotion” (Gruber and Moskowitz, 2014), as granularity seems to be an affective component that is separable from categorical meaning.

Even if we grant this distinction between enjoyment and pleasure, it remains to be demonstrated that emotional granularity can explain enjoyable sadness. The plausibility of our theory is suggested, however, by evidence that finely differentiated negative emotions are experienced as less “unpleasant” (Barrett et al., 2001; Kashdan et al., 2015; Smidt and Sudak, 2015). Although this evidence pertains only to verbal discriminations of emotions, it supports our suggestion that differentiation alters the experience of negative emotion. If Mendelssohn was right about the “preciseness” of musical emotion, then perhaps music can render negative emotions not just endurable but enjoyable. This possibility is supported by evidence that elicitation of a “multifaceted emotional experience” is correlated with the enjoyment of sad music (Taruffi and Koelsch, 2014).

## Pleasure and enjoyment in music

According to our theory it should be possible to discriminate musical pleasure from enjoyment, although the two are typically mixed together. There are a number of ways for music to give pleasure in the narrow sense; in fact much of what is studied under the rubric of musically induced emotion fits into this category: soothing textures and harmonies, rhythmic coherence and “groove,” and expressive contours or gestures (e.g., Juslin and Vastfjall, 2008). There are also diverse means for the production of musical *displeasure*—dissonance, noise, rhythmic incoherence, etc. However, because the reception of musical feelings depends on how they are embedded within the overall musical experience, reports of isolated feelings of musical displeasure may be rare. For instance, dissonance is often ingredient in enjoyable music and where it is reported as unpleasant it is usually part of a thoroughly unenjoyable experience of “bad” music (Gabrielsson, 2011).

Musical enjoyment as defined here has been theorized elsewhere (Benzon, 2001) but awaits the formulation of a more precise and testable model of its underlying dynamics. We suggest that resources for constructing such a model are emerging from studies of the musical entrainment—i.e., rhythmic synchronization—of sensorimotor dynamics (e.g., Clayton et al., 2005; Janata et al., 2012; Merchant et al., 2015) and the relationship between music perception and movement (e.g., Maes et al., 2014). Especially pertinent are arguments from ecological psychology that the phenomenon of musical motion—the experience that someone or something is moving in music—is not a metaphorical mapping but rather a direct perceptual experience of various kinds of “virtual motion” (gestures, movement within an environment, etc.) specified by dynamic features of the music itself (Clarke, 2001, 2005; Bharucha et al., 2006; Eitan and Granot, 2006). Together, these viewpoints suggest that musical stimuli can be coupled with sensorimotor processes of the brain in a manner that (1) drives widespread rhythmic coordination of neural activity and (2) overlaps with perceptual experiences of motion in a highly structured environment.

For the present thesis, the key implication is that feelings of negative emotion, when induced by music, are embedded within richly structured experiences of motion which serve to “perceptualize” the experience of emotion. Thus, for instance, a sigh-like phrasing is not just an icon of emotion but also a directly perceived manifestation of emotion. Such manifestations can be categorized in multiple ways—to say that emotions are “perceptualized” does not mean that they are simply “read off” the music. But however they are categorized, musical emotions are experienced through movement and are therefore more concretely formed than “normal” emotion. Musical motion, therefore, is what gives musical emotion its high granularity. What makes negative emotions in music enjoyable is the special way in which music moves us in a quite literal way, as indicated by the close relationship between music and dance (Schulkin, 2013; Fitch, 2016).

In short, what distinguishes the neurodynamic approach is its promise to explain how emotions are co-constituted and enriched by the perceptual experience of music (Krueger, 2014), whereas other approaches usually aim only to explain how emotions are *triggered* by music. But emotions are not just triggered by music; they are vividly rendered in animate and highly “granular” form by the rhythmic entrainment of experience.

## Prospects for further research

The central claim of our proposal is that musical enjoyment of negative emotion is distinguished by high emotional granularity in comparison with “normal” (i.e., unenjoyable) experiences of negative emotion. We see two possible ways to test this claim.

One way is to refine methods of self-reporting (Juslin and Sloboda, 2010) in an attempt to gather data about the emotional granularity of musical experiences. It should be emphasized that most published studies of emotional granularity have aimed only to measure subjects' verbal capacity to discriminate emotions (Smidt and Sudak, 2015); this capacity is, at best, an indirect measure of the actual emotional granularity of experience (see Lindquist and Barrett, 2008). Here, we are interested only in *experienced* emotional granularity; moreover we are interested in how this granularity *varies* in response to music. Self-reporting is notoriously unreliable, and continuous variations of granularity are likely to be even harder to report than categorical distinctions (e.g., happy/sad). Nevertheless, while acknowledging these limitations, we believe that self-reporting methods could be used to test our theory—as suggested by at least one study (Taruffi and Koelsch, 2014).

A second possible test would be to measure variations of emotional granularity using high temporal-resolution techniques such as EEG and MEG. In recent years, neuroscientists who adopt a neurodynamic approach have begun using these techniques to measure “moment-to-moment brain signal variability” associated with cognitive functioning (Heisz et al., 2012; Garrett et al., 2013; Miskovic et al., 2016) and at least one study has attempted to measure correlations between individual emotional granularity and brain activity during “affective processing” (Lee et al., 2017). To test our proposal what is needed is a way to test variations of granularity within the same individual in response to music, and for this it is necessary to develop a measure of emotional complexity (cf. Seth et al., 2006). If such a measure could be developed, our expectation is that experiences of musically induced negative emotion would be found to correlate with higher levels of complexity than “normally” induced (e.g., IAPS-induced) negative emotion.

At present, however, there is no evidence that directly supports our theory. Even so, we believe that the idea of emotional granularity as a factor in musical enjoyment is plausible and worthy of further investigation. Moreover, it should be noted that the thesis presented here is not just about musical enjoyment: it is also about the role of granularity in enjoyment, pleasure, and emotional experience in general. As such, its various phenomenological and neurological implications need to be further articulated and tested against a very wide array of empirical evidence. The key insight of this perspective, which fits well with a neurodynamic approach, is that emotional experiences can vary in dimensionality (coarse vs. fine) as well as categorical meaning (happy vs. sad).

## Author contributions

NB and JS made contributions to the drafting and revision of this work at all stages and share responsibility for its content.

### Conflict of interest statement

The authors declare that the research was conducted in the absence of any commercial or financial relationships that could be construed as a potential conflict of interest.
